# Supplementary material for: Guiding functions of the C-terminal domain of topoisomerase IIα advance mitotic chromosome assembly
Source: Nat Commun. 2021 May 18;12:2917. doi: 10.1038/s41467-021-23205-w (PMC8131626; doi:10.1038/s41467-021-23205-w)
Supplement: Supplementary file 1 — Supplementary Information [file 41467_2021_23205_MOESM1_ESM.pdf]

Supplementary information for

**Guiding functions of the C-terminal domain of topoisomerase IIa advance mitotic chromosome assembly**

**Keishi Shintomi and Tatsuya Hirano**

**This PDF includes:**
Supplementary Figures 1-3
Supplementary Table 1

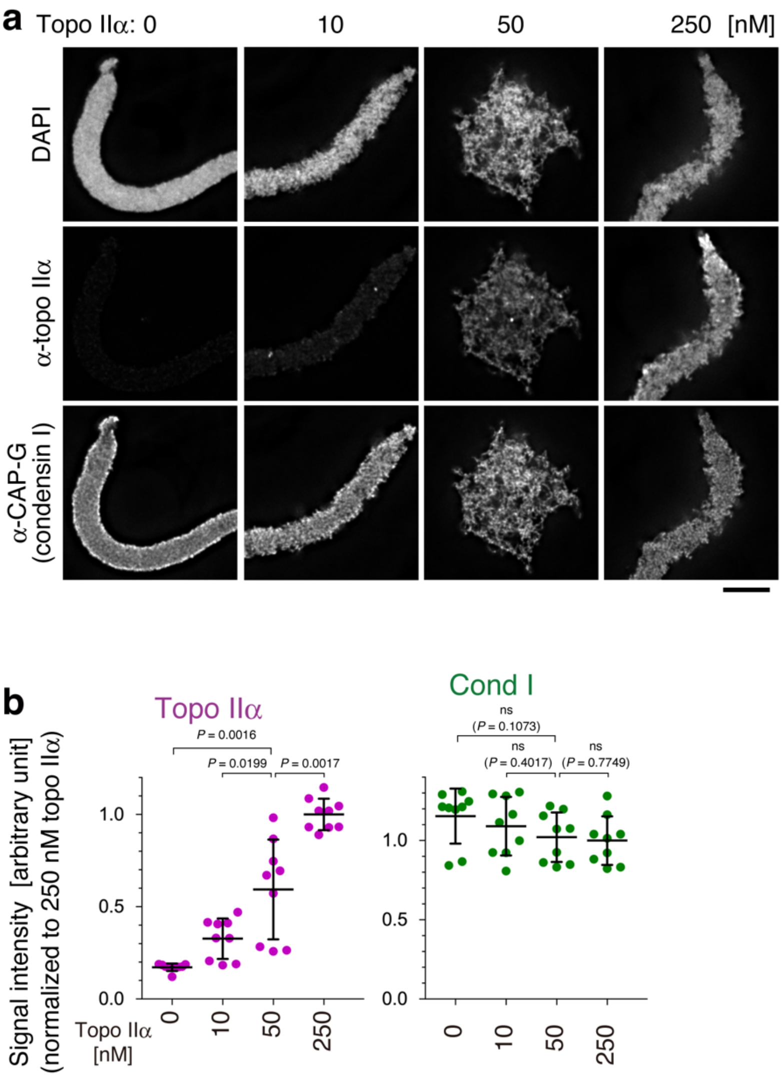


**Supplementary Fig 1**. **Titration of recombinant *Xenopus* topo IIα in the mitotic chromatid reconstitution assay. a, b.** Chromatid reconstitution assays were performed using increasing concentrations of *Xenopus* topo IIα and fixed concentrations of the other five proteins (condensin I, a truncation version of histone H2A.XF-H2B dimer, Npm2, Nap1, and FACT). After a 150-min incubation, the resultant structures were fixed and immunolabeled with antibodies against topo IIα and CAP-G. DNA was counterstained with DAPI (**a**). Quantitative analyses of the signal intensities were carried out as in Fig. 1. The mean ± s.d. is shown (*n*=9 clusters of chromatin). *P* values were assessed by two-tailed Welch's *t*-test (ns, not significant) (**b**). Bar, 5 μm.

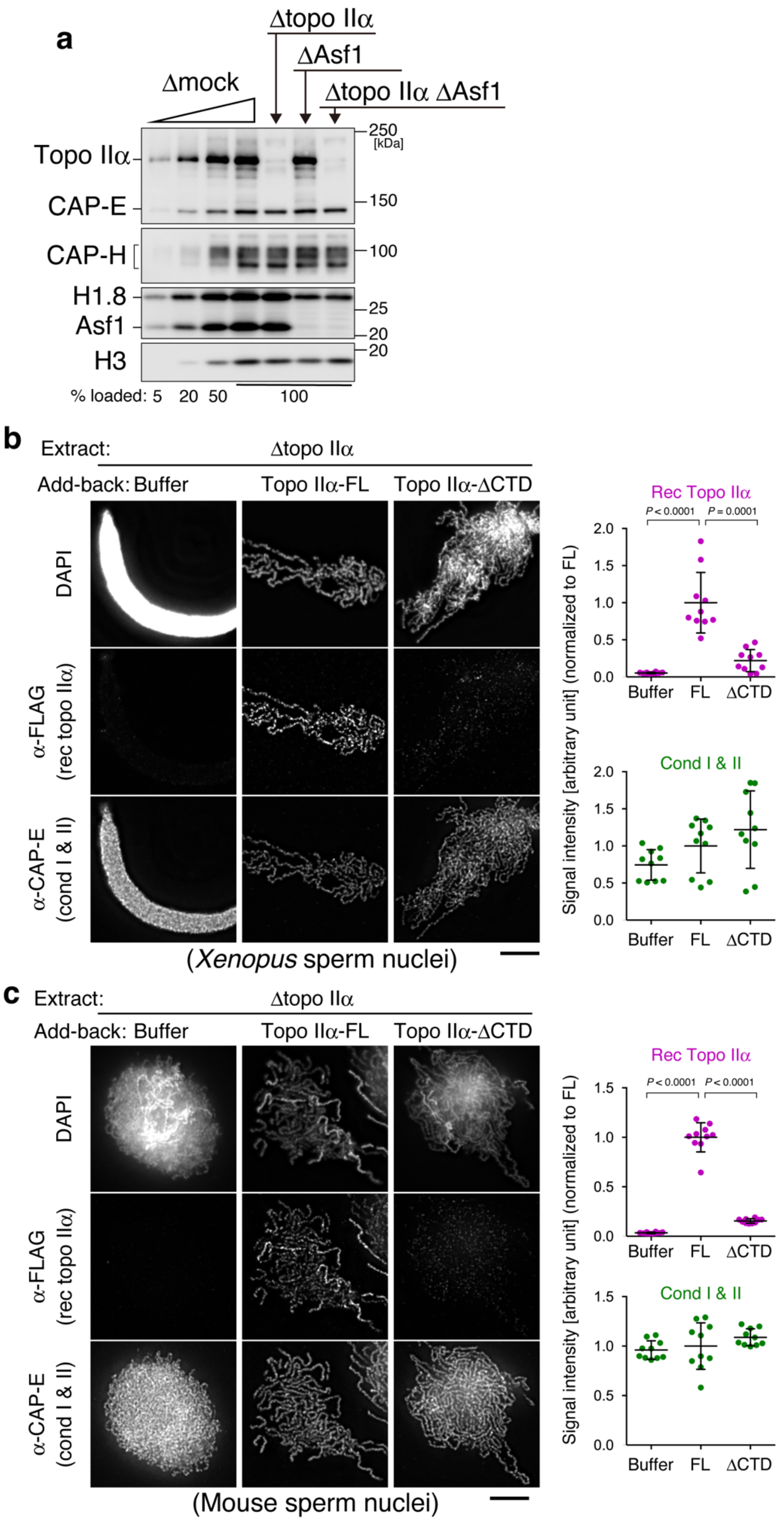
a
Δtopo IIα
ΔAsf1
Δtopo IIα ΔAsf1
Δmock
Topo IIα
CAP-E
CAP-H
H1.8
Asf1
H3
250 [kDa]
150
100
25
20
20
% loaded: 5 20 50 100
b
Extract: Δtopo IIα
Add-back: Buffer Topo IIα-FL Topo IIα-ΔCTD
DAPI
α-FLAG (rec topo IIα)
α-CAP-E (cond I & II)
(Xenopus sperm nuclei)
Rec Topo IIα
P < 0.0001 P = 0.0001
Cond I & II
Signal intensity [arbitrary unit] (normalized to FL)
Buffer FL ΔCTD
c
Extract: Δtopo IIα
Add-back: Buffer Topo IIα-FL Topo IIα-ΔCTD
DAPI
α-FLAG (rec topo IIα)
α-CAP-E (cond I & II)
(Mouse sperm nuclei)
Rec Topo IIα
P < 0.0001 P < 0.0001
Cond I & II
Signal intensity [arbitrary unit] (normalized to FL)
Buffer FL ΔCTD

**Supplementary Fig 2**. **Topo IIα-ΔCTD is proficient in chromatid individualization, but is deficient in chromatid thickening in *Xenopus* egg cell-free extracts. a.** Mitotic egg extracts were immunodepleted with a control rabbit IgG (Δmock), anti-topo IIα antibody (Δtopo IIα), ant-Asf1 antibody (ΔAsf1), or a combination of anti-topo IIα and anti-Asf1 (Δtopo IIα ΔAsf1). To estimate the efficiency of depletion, an aliquot of each extract along with decreasing amounts of the Δmock extract was analyzed by immunoblotting with the antibodies indicated. Note that neither condensin subunits (CAP-E and CAP-H) nor histone H3 was depleted after each depletion while a sizable portion (50~80%) of the linker histone H1.8 was co-depleted with anti-Asf1 antibody (i.e., in the ΔAsf1 and Δtopo IIα ΔAsf1 extracts). This experiment was repeated independently at three times with similar results. **b.** *Xenopus* sperm nuclei were added to egg extracts depleted of topo IIα (Δtopo IIα) that had been supplemented with buffer, topo IIα-FL, or topo IIα-ΔCTD. After a 150-min incubation, the samples were fixed and processed for immunofluorescence and analyzed. Quantitative analyses of the signal intensities were carried out as in Fig. 1. The mean ± s.d. is shown ($n$=12 clusters of chromatin). $P$ values were assessed by two-tailed Welch's $t$-test. **c.** Mouse sperm nuclei were used in the same experiment as shown above. The mean ± s.d. is shown ($n$=12 clusters of chromatin). $P$ values were assessed by two-tailed Welch's $t$-test. Bars, 5 µm.

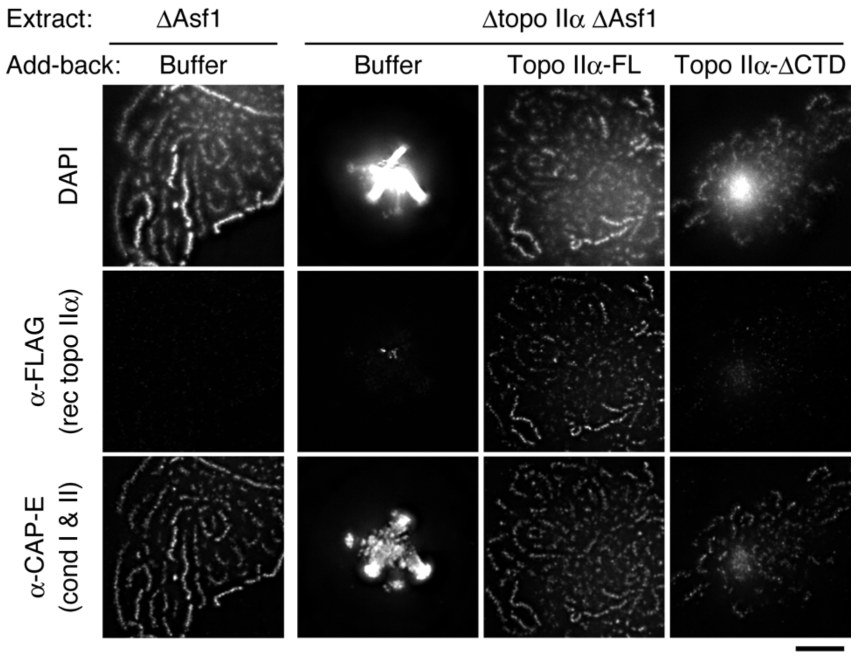


**Supplementary Fig 3**. **Topo IIα-FL, but not topo IIα-ΔCTD, can fully rescue the defects observed in the extracts depleted of both topo IIα and Asf1 (topo IIα and CAP-E labeling).** Mouse sperm nuclei were added to egg extracts depleted of topo IIα and Asf1 (Δtopo IIα ΔAsf1) that had been supplemented with buffer, topo IIα-FL, or topo IIα-ΔCTD. After a 150-min incubation, the samples were fixed and processed for immunolabeling with the antibodies indicated. As a reference, nucleosome-depleted chromatids were assembled in an extract depleted of Asf1 (ΔAsf1) that contained endogenous topo IIα. This experiment was repeated independently at three times with similar results. Bar, 5 µm.

**Supplementary Table 1. The list of primers used in this study.**

| Identifier | Sequence | Note |
|---|---|---|
| **KB87** | GACGATGACAAGCCCGAGACCGAACCTTTGCAGCCTC | PCR (topo IIα-FL and -ΔCTD), forward, overlapping sequence A for Gibson assembly |
| **KB88** | CAGAACTTCCAGCCCAAAGTCATCGTCAGAATCCTCCAGG | PCR (topo IIα-FL), reverse, overlapping sequence B for Gibson assembly |
| **KB89** | CAGAACTTCCAGCCCAATTTCCTGCAACTGCATTTTC | PCR (topo IIα-ΔCTD), reverse, overlapping sequence B for Gibson assembly |
| **KA28** | AAATGATAACCATCTCGC | Sequencing (pXR504, 505, and 506) |
| **KA29** | GAAATTTGTGATGCTATTGC | Sequencing (pXR504, 505, and 506) |
| **KU27** | AAGATTAAGCCATTTGAGGG | Sequencing (pXR505 and 506) |
| **KU28** | GTCGTGGCTGAGCAATACGC | Sequencing (pXR505) |
| **KU31** | CAACATCATCAAGATCGTGGG | Sequencing (pXR505 and 506) |
| **KU32** | TTCCAAAATGAATCTGGCTTGG | Sequencing (pXR505 and 506) |
| **KU33** | ATGTCTGCATACCATCATGGTG | Sequencing (pXR505 and 506) |
| **KU34** | TGTCGAGCACAGACACCTCACC | Sequencing (pXR505 and 506) |
| **KU35** | GTAAAGAAAATGCAGTTGCAGG | Sequencing (pXR505 and 506) |
| **KU36** | CTTTATGCAGGACATAGTTGGG | Sequencing (pXR505 and 506) |
| **KU37** | GATACTCCCATCATGATCAGTGC | Sequencing (pXR505) |
| **KB90** | GAGTACCACACTGATACCAC | Sequencing (pXR505 and 506) |
| **KB91** | TCGCTTCTTGGCTGCTGCAG | Sequencing (pXR505) |
| **KB92** | CCCTGCGGTCTTGCATGAAG | Sequencing (pXR505 and 506) |
| **KB93** | GTGGGAATACACCATAGCG | Sequencing (pXR505 and 506) |
| **KB94** | GATCATTCACCTGCTCGTGC | Sequencing (pXR505 and 506) |
