# Supplementary material for: Guiding functions of the C-terminal domain of topoisomerase IIα advance mitotic chromosome assembly
Source: Nat Commun. 2021 May 18;12:2917. doi: 10.1038/s41467-021-23205-w (PMC8131626; doi:10.1038/s41467-021-23205-w)
Supplement: Supplementary file 3 — Reporting Summary [file 41467_2021_23205_MOESM3_ESM.pdf]

nature research

Corresponding author(s): Tatsuya Hirano
Last updated by author(s): Mar 26, 2021

Reporting Summary

Nature Research wishes to improve the reproducibility of the work that we publish. This form provides structure for consistency and transparency in reporting. For further information on Nature Research policies, see our Editorial Policies and the Editorial Policy Checklist.

Statistics

For all statistical analyses, confirm that the following items are present in the figure legend, table legend, main text, or Methods section.

n/a	Confirmed	
☐	☒	The exact sample size (n) for each experimental group/condition, given as a discrete number and unit of measurement
☐	☒	A statement on whether measurements were taken from distinct samples or whether the same sample was measured repeatedly
☐	☒	The statistical test(s) used AND whether they are one- or two-sided<br>*Only common tests should be described solely by name; describe more complex techniques in the Methods section.*
☒	☐	A description of all covariates tested
☒	☐	A description of any assumptions or corrections, such as tests of normality and adjustment for multiple comparisons
☐	☒	A full description of the statistical parameters including central tendency (e.g. means) or other basic estimates (e.g. regression coefficient) AND variation (e.g. standard deviation) or associated estimates of uncertainty (e.g. confidence intervals)
☐	☒	For null hypothesis testing, the test statistic (e.g. F, t, r) with confidence intervals, effect sizes, degrees of freedom and P value noted<br>*Give P values as exact values whenever suitable.*
☒	☐	For Bayesian analysis, information on the choice of priors and Markov chain Monte Carlo settings
☒	☐	For hierarchical and complex designs, identification of the appropriate level for tests and full reporting of outcomes
☒	☐	Estimates of effect sizes (e.g. Cohen's d, Pearson's r), indicating how they were calculated

Our web collection on statistics for biologists contains articles on many of the points above.

Software and code

Policy information about availability of computer code

Data collection	SoftWoRx (version 7.0.0; Cytiva),<br>Amersham Imager AI680 software (version 2.0.0; Cytiva)
Data analysis	ImageJ (version 2.0.0-rc-43/1.52n)<br>Excel for Mac (version 16.x; Microsoft)<br>Prism (version 7 and 9; GraphPad Software)

For manuscripts utilizing custom algorithms or software that are central to the research but not yet described in published literature, software must be made available to editors and reviewers. We strongly encourage code deposition in a community repository (e.g. GitHub). See the Nature Research guidelines for submitting code & software for further information.

Data

Policy information about availability of data

All manuscripts must include a data availability statement. This statement should provide the following information, where applicable:

- Accession codes, unique identifiers, or web links for publicly available datasets
- A list of figures that have associated raw data
- A description of any restrictions on data availability

Source data for all statistical analyses (Figs 1b,d,e,g,h, 2d,e,g, 3b,d, 4b, 5d,f and Supplementary figs 1b, 2b,c) and uncropped image of gel and blot (Figs 2b, 3a,c and Supplementary figs 2a) are provided in the Source Data file. All other data that support the finding of this study are available from the corresponding author on reasonable request.

Field-specific reporting

Please select the one below that is the best fit for your research. If you are not sure, read the appropriate sections before making your selection.

☒ Life sciences ☐ Behavioural & social sciences ☐ Ecological, evolutionary & environmental sciences

For a reference copy of the document with all sections, see nature.com/documents/nr-reporting-summary-flat.pdf

Life sciences study design

All studies must disclose on these points even when the disclosure is negative.

Sample size	No statistical methods were used to predetermine sample size. Sufficient sample size and the number of replicates were chosen for each experiment based on other studies with similar methodology (Xue et al, 2013 [PMID: 24075807]; Zerhut et al, 2014[24952593]; Yoshida et al, 2016 [27325792]; French et al, 2017 [28743005]; Brownlee and Heald, 2019 [30639102]) and stated in figure legends.
Data exclusions	No data were excluded.
Replication	All experiments were replicated and results were reproduced at least three times. The representative results, out of there independent experiments, are shown.
Randomization	To make systematic errors as minimum as possible, we did randomization procedures as follows: All samples were allocated into experimental groups which were directly compared; Image acquisition and quantitative measurement were performed in random order; Animals were randomly selected from our colony for preparation of egg extracts and sperm nuclei.
Blinding	Blinding was not possible because experiments and data analyses were performed by the same author.

Reporting for specific materials, systems and methods

We require information from authors about some types of materials, experimental systems and methods used in many studies. Here, indicate whether each material, system or method listed is relevant to your study. If you are not sure if a list item applies to your research, read the appropriate section before selecting a response.

Materials & experimental systems

n/a	Involved in the study	
☐	☒	Antibodies
☐	☒	Eukaryotic cell lines
☒	☐	Palaeontology and archaeology
☐	☒	Animals and other organisms
☒	☐	Human research participants
☒	☐	Clinical data
☒	☐	Dual use research of concern

Methods

n/a	Involved in the study	
☒	☐	ChIP-seq
☒	☐	Flow cytometry
☒	☐	MRI-based neuroimaging

Antibodies

Antibodies used

Primary antibodies
1. anti-topo IIalpha (Hirano and Mitchison 1993, serum #alphaC1-6, used at 1:2000 dilution for both immunoblotting [IB] and immunofluorescence [IF])
2. anti-CAP-E (Hirano and Mitchison, 1994, affinity-purified #AfR9-4, used at 2.0 μg/ml for both IB and IF)
3. anti-CAP-G (Hirano et al 1997, affinity-purified #AfR11-3, used at 2.0 μg/ml for both IB and IF)
4. anti-CAP-H2 (Ono et al 2003, affinity-purified #AfR201-4, used at 2.0 μg/ml for both IB and IF)
5. anti-Asf1 (Shintomi et al 2017, serum #R461-4, used at 1:2000 dilution for IB)
6. anti-H1.8/B4 (Shintomi et al 2005, serum #116-5, used at 1:2000 dilution for both IB and IF)
7. anti-H3 (ab1791 [RRID:AB_302613], Abcam, used at 0.5 μg/ml for IB)
8. anti-H3 (CMA301 [RRID:AB_1977240], MBL, used at 2.0 μg/ml for IF)
9. anti-FLAG M2 (F-1804 [RRID:AB_262044], Sigma, used at 0.5 μg/ml for IB and 2.0 μg/ml for IF)

Secondary antibodies
10. Alexa Fluor 488 anti-mouse IgG (A11001[RRID:AB_2534069], Thermo Fisher Scientific, used at 2.0 μg/ml for IF)
11. Alexa Fluor 568 anti-rabbit IgG (A11036 [RRID:AB_10563566], Thermo Fisher Scientific, used at 2.0 μg/ml for IF)
12. horseradish peroxidase-conjugated anti-rabbit IgG (PI-1000 [RRID:AB_2336198], Vector Laboratories, used at 0.5 μg/ml for IB)
13. horseradish peroxidase-conjugated anti-mouse IgG (PI-2000 [RRID:AB_2336177], Vector Laboratories, used at 0.5 μg/ml for IB)

Validation

For lab-made antibodies (#1-6), their specificity was determined by IB analysis of Xenopus egg extracts. All of them mainly recognized bands that migrate to estimated positions on SDS-PAGE. It was also confirmed these bands were completely diminished upon

depletion of corresponding proteins from the extracts. For antibodies for histone H3 (#8 and 9), thier specificity was determined by IB and IF analyses of chromatin assembled in egg extracts. These signals were confirmed to be diminished upon depletion of the H3 chaperone Asf1 (Shintomi et al, 2017).

Eukaryotic cell lines

Policy information about cell lines

Cell line source(s)	The Sf9 cell line (isolated from ovaries of the armyworm Spodotera frugiperda) for production of recombinant proteins were purchased from Thermo Fisher Scientific.
Authentication	The Sf9 cell line were authenticated by karyotyping by the vendor.
Mycoplasma contamination	No tests for mycoplasma contamination were performed.
Commonly misidentified lines (See ICLAC register)	No commonly misidentified lines were used.

Animals and other organisms

Policy information about studies involving animals; ARRIVE guidelines recommended for reporting animal research

Laboratory animals	The female (2-4 years old) and male (1.5-2 years old) frogs of Xenopus laevis (whose strains were not determined) were used for preparation of egg extracts and sperm nuclei; The male mice (10-20 weeks old) of Mus musculus (the F1 hybrids of BALB/c and C57BL/6J) were used for preparation of sperm nuclei.
Wild animals	This study does not involve any wild animals.
Field-collected samples	This study does not involve any field-collected animals.
Ethics oversight	All animals were used in compliance with the institutional regulations and expeRiments were approved with the Animal Experimental Committee of the RIKEN Wako InstItute.

Note that full information on the approval of the study protocol must also be provided in the manuscript.
